# 

**Published:** 1921-01-22

**Authors:** 


### Gifts and Bequests.

The ultimate residue of the estate of Mrs. Marianne Rowe, of Folkestone, has been left to the Royal London Ophthalmic Hospital.

The Royal Victoria and Mater Infirmorum Hospitals at Belfast have each received £2,500 from the Committee of the Hospital Sports Gala.

Major E. A. F. W. Herbert, late Yorkshire Hussars, has left £100 to the York County Hospital and £50 to the Montgomeryshire Infirmary.

The residents of Marylebone have raised 6,000 guineas to endow in perpetuity six beds in the Middlesex Hospital.

The Wolverhampton and District Hospital for Women and three other institutions receive £50 each under the will of the late Alderman F. H. Skidmore.

The proceeds of the disposal by auction of the estate of the late Mr. F. S. Goddard, of Farnborough, amounted to £3,000, and have been handed to Guildford Hospital as required by the will.

### Personalia.

The King has approved the appointment of Mr. S. P. Vivian, a Principal Assistant Secretary in the Ministry of Health, and Deputy Registrar-General, to be Registrar-General in succession to Sir Bernard Mallet, K.C.B.

In recognition of services rendered in the war, the Rev. A. Lombardini, Chaplain of Kensington Infirmary, has been appointed by the War Office an Honorary Chaplain to the Forces.

Miss Irene T. J. Ruxton, M.B., Ch.B., Aberdeen, has been appointed assistant school medical officer at Newport (Mon.), at a salary of £650 per annum.

Miss Mabel Hughes, assistant matron at the Great Ormond Street Hospital for Children, London, who had the honour of training H.R.H. Princess Mary in nursing, was married on January 5, at Gelligaer, South Wales, to Dr. D. J. Thomas, medical officer of health to the Gelligaer Urban District Council and Chairman of the local bench of magistrates.

### Forthcoming Events.

Thursday, January 27.—The staff dinner, followed by a dance, of the Dental Branch of University College Hospital will be held at the Hotel Cecil. The music will be supplied by the Boston Jazz Band.

The Chelsea Football and Athletic Co., Ltd., have sent a donation of twenty-five guineas, and the Fulham Football and Athletic Co., Ltd., a donation of ten pounds to the Royal Hospital and Home for Incurables, Putney. These amounts are part of the proceeds of charity matches.

### Matrons' and Sisters' Appointments.

Metropolitan Asylums Board Hospital, Sheffield Street, Kingsway.—Miss G. M. W. Nash has been appointed superintendent sister. She was trained at the Croydon Infirmary and Queen Charlotte's Hospital, becoming subsequently sister and then assistant matron of the former institution. She has also served as sister under the Q.A.I.M.N.S.R. and as night sister at the Royal Free Hospital.

Infectious Diseases Hospital, Blackpool.—Miss Kate Amy has been appointed sister. She was trained at the Royal Victoria Infirmary, Belfast, and the City Hospital, Fazakerley, Liverpool, and has since been engaged in private nursing.

Kaimshill Sanatorium, Kilmarnock.—Miss Marion J. MacAlister has been appointed matron. She was trained at Belvidere Fever Hospital, Glasgow, and at the Glasgow Royal Infirmary, six years in all. In 1918 she was sister-in-charge of the *x*-ray department of the Scottish Women's Hospital at Royaumont, and later became sister at the Stewartry Infectious Diseases Hospital, Castle Douglas, Kirkcudbrightshire.

Sculcoates Union Infirmary, Beverley Road, Hull.—Miss Annie Eliza Magdalen Gillman Watson has been appointed night sister. She was trained at the Royal Free Hospital, the North Western Hospital, Haverstock Hill, and at the Mothers' Hospital, Clapton. She has since been staff nurse and sister at the Coventry and Warwickshire Hospital, has done private nursing, and has been superintendent nurse at Cockermouth Union Hospital.

Cameron Hospital, West Hartlepool.—Miss L. M. Naylor has been appointed sister. She was trained at York County Hospital.

Accident Hospital, Longton, Staffs.—Miss E. Williams has been appointed theatre sister. She was trained at the General Infirmary, Wrexham, and has since been sister at Bedford County Hospital, and Darlington General Hospital.

Udal Torre Sanatorium, Yelverton.—Miss Ethel Margaret Henderson has been appointed matron. She was trained at University College Hospital, London, and has since been sister of the women's medical ward at the Norfolk and Norwich Hospital, sister and assistant matron at Kelling Sanatorium; she has also engaged in private nursing and under the Q.A.I.M.N.S.R.

Horton Infirmary, Banbury.—Miss W. M. L. Abel has been appointed night sister. She was trained at Greenock Infirmary and at Campbell Hospital, Portsoy, Scotland, and served with the Q.A.I.M.N.S.R. from 1917 till the beginning of this year.

Victoria Hospital, Blackpool.—Miss Mary Wilding has been appointed theatre sister. She was trained at this hospital, where she has been holiday sister. She has also held the post of sister at Longton Accident Hospital.

New End Hospital, Hampstead.—Miss M. W. Gallant has been appointed sister. She was trained at the Poplar and Stepney Sick Asylum and the East End Mothers' Home, and has held the following posts: Sister at the Endell Street Military Hospital, staff nurse and sister Q.A.I.M.N.S.R., and sister under the British Committee of the Russian Red Cross.

Infants' Hospital, Vincent Square, S.W.—Miss Edith Clegg has been appointed night sister. She was trained at the Crossland Moor Hospital, Huddersfield, where she was later sister. She has held the post of sister at the Women's Settlement Hospital, Plaistow, and been engaged in private nursing. She holds the Central Midwives Board certificate.

Seamen's Hospital, Greenwich.—Miss Elizabeth Dunn has been appointed theatre sister. She was trained at the Royal Infirmary, Manchester, where she was later theatre sister and out-patient sister. She has also held the posts of ward sister, theatre sister, and night superintendent under the Q.A.I.M.N.S.

